# Ocular Safety and Toxicology of Subretinal Gene Therapy With rAAV.hPDE6A in Nonhuman Primates

**DOI:** 10.1167/tvst.14.1.29

**Published:** 2025-01-29

**Authors:** Immanuel P. Seitz, Fabian Wozar, Guy Alex Ochakovski, Felix F. Reichel, Sven Korte, Birgit Korbmacher, Barbara Wilhelm, Daniela Süsskind, Karl-Ulrich Bartz-Schmidt, M. Dominik Fischer, Tobias Peters, for the RD Cure Consortium

**Affiliations:** 1 University Eye Hospital Tuebingen, Centre for Ophthalmology, University of Tuebingen, Tuebingen, Germany; 2 University Eye Hospital Munich, Munich, Germany; 3 Labcorp Early Development Services GmbH, Muenster, Germany; 4 Virscio, Inc., New Haven, CT, USA; 5 STZ *eyetrial*at the Centre for Ophthalmology, Tuebingen, Germany; 6 Nuffield Department of Clinical Neurosciences, University of Oxford, Oxford, UK

**Keywords:** retinitis pigmentosa, PDE6A, gene therapy, toxicity, nonhuman primates

## Abstract

**Purpose:**

Reports of gene therapy–associated retinal atrophies and inflammation have highlighted the importance of preclinical safety assessments of adeno-associated virus (AAV) vector systems. We evaluated in nonhuman primates (NHPs) the ocular safety and toxicology of a novel AAV gene therapy targeting retinitis pigmentosa caused by mutations in *PDE6A*, which has since been used in a phase I/II clinical trial (NCT04611503).

**Methods:**

A total of 34 healthy cynomolgus animals (*Macaca fascicularis*) were treated with subretinal injections of rAAV.hPDE6A and followed over 13 weeks. Three dose levels (low: 1 × 10^11^, intermediate: 5 × 10^11^, and high: 1 × 10^12^ vector genomes [vg]) were compared to sham-injected controls. Safety and toxicity were determined using ophthalmic examinations, electroretinography, ocular histology, and retinal imaging.

**Results:**

At the low and intermediate doses, inflammation was mild, electroretinography response was unimpeded, and histology results were in line with surgically induced changes. In contrast, three high-dose animals displayed atrophic changes of the retina and abnormalities in electroretinography, which were considered test article related and adverse.

**Conclusions:**

A single subretinal injection of up to 5 × 10^11^ vg was well tolerated, and a 10-fold lower dose of 5 × 10^10^ vg was chosen as the starting dose for the ongoing phase I/II clinical trial. Atrophic retinal changes and abnormalities in electroretinography emerged as dose-limiting findings in the high-dose cohort.

**Translational Relevance:**

This study demonstrates that treatment candidate rAAV.PDE6A was well tolerated in NHPs. Occurrence of retinal atrophy as a dose-limiting finding highlights the importance of further study into the mechanisms of atrophy induction after retinal gene therapy.

## Introduction

In light of recent events of retinal atrophy following subretinal gene augmentation therapy for biallelic RPE65 mutations^1^^,^^2^ and severe cases of gene therapy–associated inflammation after intravitreal gene therapy for diabetic retinopathy, safety considerations seem increasingly relevant to clinical ocular gene therapy (GT) trials. Given that adeno-associated virus (AAV) is still the dominant vector system and continues to evolve,^3^^,^^4^ and viable alternatives to it still need to mature further,^5^ these safety concerns seem bound to remain relevant for years to come. The fact that the next generation of CRISPR-based gene therapies relies on molecules of bacterial origin and is often delivered by AAV vectors will also not help the issue.^6^^–^^8^ Even though not all adverse reactions can be anticipated in preclinical trials, the current situation underlines the importance of comprehensive preclinical safety testing and dose finding in large animal models, preferably nonhuman primates (NHPs), which are immunologically most similar to humans, prior to attempts of clinical translation.^9^

Retinitis pigmentosa (RP) denotes a family of inherited retinal dystrophies characterized by progressive retinal degeneration that usually starts in the periphery and progressively expands centripetally. Accordingly, patients experience a sequence of visual deteriorations that start with poor night vision, often already in the first or second decade of life, which progresses into loss of peripheral vision (tunnel vision) in the third and fourth decades and eventually causes loss of central high-acuity vision, which in many cases may lead to near-complete blindness at late stages. The prevalence of RP is approximately 1:4000.^10^ RP is genetically very heterogeneous, with 20% to 30% of cases being autosomal recessively inherited (arRP). The most prevalent genes mutated in arRP are EYS (5%–12%),^11^^,^^12^ USH2A (5%–15%),^13^^,^^14^ CRB1 (∼5%),^15^ and PDE6B (4%–10%),^16^ but these numbers largely depend on the population studied. Mutations in *PDE6A*, encoding the alpha subunit of the rod photoreceptor cGMP phosphodiesterase (RP43 locus), are reported to be around 2% to 4% of arRP cases.^17^ This puts the absolute number of patients estimated to be affected with *PDE6A*-linked arRP in the European Union at about 1200 to 3100. As with all types of RP, except for those caused by biallelic RPE65 mutations, no treatment is available for retinal disease caused by mutations in *PDE6A*. In 2019, the University of Tübingen began conducting the world's first phase I/II clinical trial for *PDE6A*-related RP. This publication details the results of two consecutive no-human primate trials, which were performed to evaluate the safety and toxicology of rAAV2/8.hPDE6A in preparation for the clinical trial and establish the vector dose to be used in humans. rAAV2/8.hPDE6A is a recombinant adeno-associated virus (rAAV), expressing the human *PDE6A* complementary DNA (cDNA) under control of a human rhodopsin (RHO) promoter. Previously, gene replacement using this vector had been shown to be effective in a mouse model homozygous for a pathogenic variant in *PDE6A*, with expression of the PDE6A protein for up to 6 months postinjection and a rescue of retinal morphology.^18^^,^^19^ Furthermore, safety and efficacy of the vector have been demonstrated in dogs.^20^^–^^22^

## Methods

### Study Design

Two consecutive NHP studies were conducted in which a total of 34 animals were treated with a single subretinal injection and observed over a 13-week period. Three dose levels (low: 1 × 10^11^, intermediate: 5 × 10^11^, and high: 1 × 10^12^ vector genomes [vg]) were compared to sham-injected (balanced saline solution [BSS]) controls. For the first study (study A), 22 animals were divided into three gender-balanced groups. Six animals served as sham-injected controls. Eight animals received a low dose (1 × 10^11^ vg), and eight further animals received a high dose of the test article (1 × 10^12^ vg). In study A, only one eye (left side) was treated in all groups, with the untreated eyes serving as an internal control. For the second study (study B), 18 animals were divided into three groups of six animals, which were used to expand ocular safety data on the sham, low-dose, and high-dose regimens. In addition, an intermediate dose level (5 × 10^11^ vg) was introduced. Here, one eye (again left side) was treated with the test article, and the contralateral eye was used for sham injections.

### Surgery and Postsurgical Care

The test article was administered by sutureless 23-gauge vitrectomy combined with a subretinal injection. Treated eyes were dosed once with a dose volume of up to 170 µL. Sham eyes were treated with the same vehicle that was used to dilute vector solutions: balanced sterile saline solution for intraocular irrigation (Industria Farmaceutica Galenica Senese Ref: DD0412004, Supp: Beaver-Visitec Intl. Ref: 581732) with 0.001% Pluronic. The same disposable supplies were used for all surgeries. For subretinal injection, an extendible 41-gauge subretinal injection needle (23/41 gauge) was used (DORC 1270.EXT). Injection pressures were controlled by a preset range at the level of the vitrectomy machine (14–52 kPa) and similar for all groups. Blebs were raised temporal of the optic nerve head, along the superior vascular arcades. Small BSS preblebs were raised in AAV-treated eyes to reduce spillover of vector solution into the vitreous cavity while raising the bleb. No significant reflux was noted in any of the treated eyes. After the injection procedure, 2 mg subconjunctival dexamethasone was applied. Animals also received 1% prednisolone eye drops three times a day for 1 week and prednisone 1 mg/kg intramuscular from postoperative days 2 to 5.

### Ophthalmoscopy and Intraocular Pressure

Ocular safety was evaluated using ophthalmic examination, including funduscopy, slit-lamp examinations, and intraocular pressure (IOP) measurements. These were performed at baseline, day 3 ± 1, day 7 ± 1, week 4 or 5, and week 12 or 13 by a team of two expert veterinarians with longstanding experience in ophthalmologic examinations in nonhuman primates. At week 4, additional cross-validation was performed by an external, masked ophthalmologist. Reported parameters from these examinations include anterior chamber cells (ACCs), anterior chamber hemorrhage (ACH), vitreous hemorrhage (VH), and suspect fundus findings such as hemorrhages at the injection site. ACCs were graded semiquantitatively from 0 to 4+ according to the Standardization of Uveitis Nomenclature working group grading scheme. ACH and VH was graded analogous to ACC. IOP measurements are reported as mm Hg.

### Electroretinography

Single-flash electroretinography (ERG) was performed using the Nicolet Viking ERG system (Nicolet Biomedical Instruments, Madison, WI, USA) in study A only. Measurements were recorded once at baseline and at weeks 4 and 12 after dosing. Electrodes (differential, indifferent, and corneal: ERG jet contact lens electrode and subdermal platinum/iridium needle electrode from Nicolet Biomedical Instruments) were applied and control measurements performed to ensure a proper fit. Dark adaption was performed for 30 minutes. Then, the following measurements were recorded for both eyes: scotopic ERGs, including six measurements (white flash, rod response) with increasing flash intensities (0.0095, 0.03, 0.095, 0.3, 3.0, and 9.49 cds/m^2^) up to the standard flash (SF) of 10 cds/m^2^ and no background luminance, followed by photopic ERG: white flash response to the standard flash of 3.0 cds/m^2^ and background luminance 100 cds/m^2^. Due to a technical issue, rod-specific scotopic ERG recordings at low flash intensities were limited to the b-wave only.

### Ocular Imaging

Fundus autofluorescence (AF), optical coherence tomography (OCT), fluorescein angiography (FA), and indocyanine green angiography were performed at baseline, week 2, week 4, week 8, and week 12 (end of observation) using a Heidelberg Engineering (Heidelberg, Germany) FLEX OCT device. A quantitative analysis of the imaging, including a detailed description of the methodology, has been published previously.^23^

### Histology

After necropsy, treated and untreated eyes underwent histopathology. The 12-o'clock position of each eye was marked prior to enucleation. Eye cups (posterior segments) were treated with 4% paraformaldehyde solution for 24 hours and then dehydrated using ascending concentrations of sucrose (10%, 20%, and 30% each for 2 hours; sucrose [e.g., SIGMA S0389], diluted in 0.15 M phosphate-buffered saline [PBS] without Ca^2+^/Mg^2+^, pH 7.3). Tissues were then embedded in optimal cutting temperature compound and frozen on dry ice–cooled isopentane for 1 hour. Using the scleral marking, globes were then oriented as to start vertical sectioning from the nasal side. Sections nasal to the optic nerve head were discarded. From the midsection of the optic nerve head onward, every 80 µm, a vertical cryosection of the whole eye cup (ora–posterior pole–ora) was mounted on SuperFrostPlus glass slides (Menzel, Braunschweig, Germany). A total of 4 mm distance from the optic nerve head toward the central macula was covered in this fashion. Slides for anatomic evaluation were then stained with hematoxylin and eosin and underwent evaluation by two independent, masked, ophthalmopathology experts under a light microscope.

### Animals

The studies were conducted at the Labcorp Early Development Services GmbH facility in Münster, Germany, in adherence to directive 2010/63/EU for the protection of animals used for scientific purposes and the 2007/526/EC Commission Recommendation (Appendix A of Convention ETS 123). The studies were in compliance with the German Animal Welfare Act and approved by the local Institutional Animal Care and Use Committee (LANUV). The procedures were compliant with the ARVO Statement for the Use of Animals in Ophthalmic and Vision Research. All animals were required to be at least 3 years of age. The sex ratio was 1:1 (male/female).

### Statistical Analysis

Statistical analysis was performed using JMP 16.2.0 (SAS Institute, Cary, NC, USA). Due to the equivalence of procedures, the results of both studies were pooled for statistical analysis. Unless specified otherwise, comparisons of treatment group means are reported as one-way analysis of variance *P* values for continuous variables and Kruskal–Wallis *H*-test *P* values for ordinal variables (such as ACC, ACH, VH). Longitudinal intragroup changes over time were analyzed using Dunnett's test with baseline measurements defined as control. To compare treatment group means for ordinal variables (such as ACC, ACH, VH), Kruskal–Wallis *H*-tests were performed.

## Results

### Ophthalmic Examination—Anterior Chamber and Vitreous Body

Following subretinal injections, 60% of treated eyes exhibited some degree of inflammatory ACCs over the 13-week observation period. Overall, incidence of ACCs was similar for all treatment groups (i.e., sham: 66%, low dose: 50%, intermediate dose: 75%, and high dose: 62%). Statistical analysis did not reveal significant differences in ACC scores between sham, low-dose, intermediate-dose, and high-dose groups. No inflammatory response in the anterior chamber was observed in untreated eyes. ACC scores peaked on day 3 ± 1 in all dose groups except for the intermediate dose, which peaked on day 7 ± 1. After day 7 ± 1, all inflammatory anterior chamber cellular activity across all groups had subsided completely.

**Figure 1.**
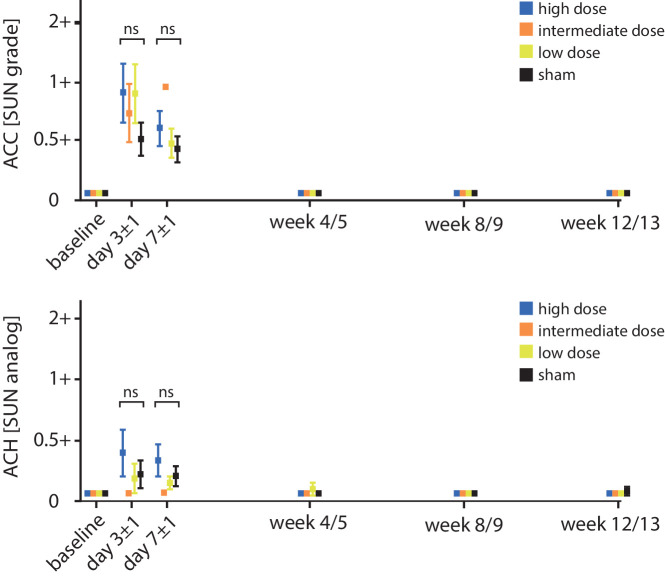
ACCs (Standardization of Uveitis Nomenclature [SUN] grade) and ACH (SUN analog) over time by dose group: *points and error bars*: mean ± SEM. ACCs were seen in 60% of all treated eyes and across all treatment regimens (including sham). After day 7 ± 1, all ACC and ACH findings had receded to baseline. ACH followed a similar trajectory, with slightly higher ACH scores in high-dose eyes. Differences between treatment groups were not statistically significant at any time point. After day 7 ± 1, all ACC and ACH findings had receded to baseline. ns, not significant, *P* > 0.05. x-axis not to scale (day 3 ± 1, day 7 ± 1).

Mild ACH was noted in up to 19% of treated eyes following surgery. By treatment group, ACH occurred in 17% of sham eyes, 17% of low-dose eyes, and 31% of high-dose eyes. No ACH was seen in intermediate-dose and untreated eyes. There was a significant (*P* = 0.0001) correlation between lower IOP and ACH severity. ACH scores peaked on day 3 ± 1 for all dose groups and then rapidly declined from there. As with ACCs, statistical analysis did not reveal significant differences in ACH scores over time between the treatment groups. After day 7 ± 1, only trace amounts of prior ACH were noted. Anterior chamber findings are summarized in Figure 1.

Inflammatory cellular activity in the vitreous chamber (vitreous cells [VCs]) was observed in 17% of sham, 8% of low-dose, and 31% of high-dose eyes. Grading was performed analogous to anterior chamber cells. VC scores peaked at day 3 ± 1 for low- and high-dose eyes and day 7 ± 1 in sham-injected eyes. VC scores were generally low, and while there was a tendency toward slightly elevated VC scores at day 3 ± 1 in the high-dose group, statistical analysis revealed no significant differences between treatment groups.

### Ophthalmic Examination—IOP and Surgical Complications

**Figure 2.**
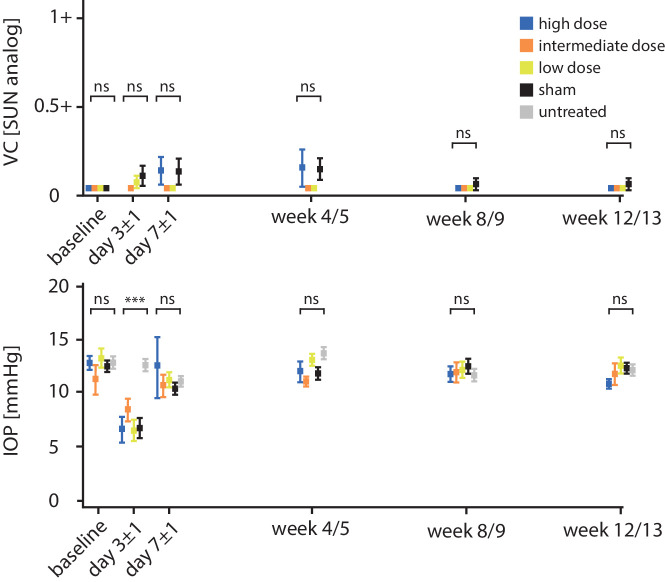
IOP (mm Hg) and vitreous cells (SUN analog) over time by dose group: *points and error bars*: mean ± SEM. All dose groups that underwent surgery displayed a significant reduction in IOP at day 3 ± 1, which had largely recovered by day 7 ± 1. No significant change in IOP was seen in eyes that remained untreated. Differences between groups were statistically significant only at day 3 ± 1. ns, not significant, *P* > 0.05; ***P < 0.0005. x-axis not to scale (day 3 ± 1, day 7 ± 1).

At day 3 ± 1, there was a significant decrease IOP in all eyes that underwent surgery compared to baseline values (sham, *P* *=* 0.0001; low dose, *P* *=* 0.0062; high dose, *P* *=* 0.0018, all treated versus untreated eyes for day 3 ± 1: *P* *=* 0.0001), while no changes in IOP were detected in untreated eyes and intermediate-dose eyes. This decline was transient. IOP had returned to baseline values in all cohorts by day 7 ± 1, except for sham-injected (*P* = 0.0002) and low-dose animals (*P* = 0.0358) in which normalization of IOP was confirmed at the subsequent time point (week 4/5). Over the complete course of observation spanning 13 weeks, there were no further anomalies regarding IOP. Figure 2 summarizes the development of vitreous cellular activity and IOP over time. Regarding surgical complications, transient intraretinal hemorrhages at the injection site, which was used for transretinal vector delivery, were described in 18 of 46 treated eyes (39.1%). Furthermore, after surgery, 3 of 46 treated eyes displayed OCT findings, which were in line with resolved macular holes (6.5%). Overall, surgical complications were evenly distributed among all treatment groups.

### Electroretinography

ERG to evaluate photoreceptor function was performed at baseline, week 4/5, and week 12/13, with *n* = 6 for sham eyes, *n* = 8 for low-dose eyes, *n* = 8 for high-dose eyes, and *n* = 22 contralateral untreated eyes serving as controls.

**Figure 3.**
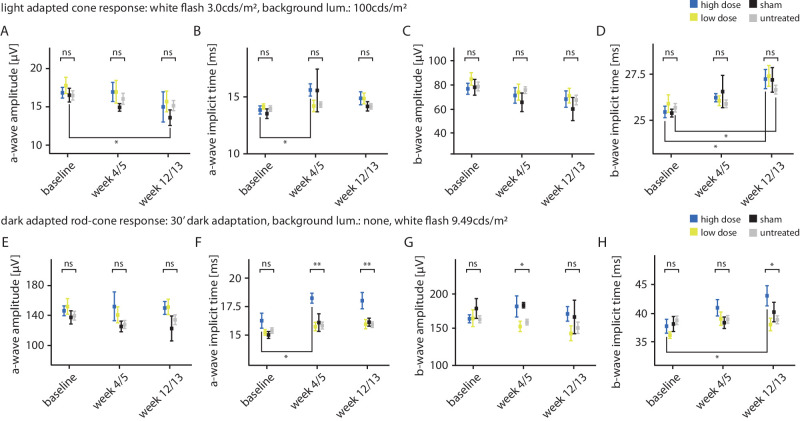
Electroretinography. The a- and b-wave amplitudes (µV) and implicit times (ms) over time by treatment group and under two different protocols: cone response (**A**–**D**) and mixed rod-cone response (**E**–**H**). *Points and error bars*: mean ± SEM. Regarding amplitudes (**A**, **C**, **E**, **G**), there were no significant differences between treatment groups at any time point, with the exception of a transient dissociation of group means in the dark-adapted mixed rod-cone response at week 4/5 (**G**) and an apparent isolated decrease of photopic a-wave amplitudes in sham-treated eyes only (**A**), which both were deemed not relevant to safety. Regarding implicit times, no significant difference between treatment groups was detected under cone-specific conditions (**B**, **D**). Yet, there was a significant increase in implicit times in high-dose-treated eyes compared to baseline. This was also noted in the dark-adapted enhanced rod-cone response. Here, significant increases in implicit times in high-dose-treated eyes compared to baseline caused a significant dissociation of group means after treatment in both a- and b-waves (**F**, **H**). In contrast to sparse other findings without correlation, such as an isolated increase of implicit times in untreated eyes (**D**), the increase in implicit times was reproduced in both a-wave and b-waves and both protocols. As they were also consistent with morphology (atrophic changes in high-dose eyes), they were therefore considered clinically significant and adverse. No remarkable findings were observed for low-dose-treated eyes under any protocol at any time point. ns, not significant, *P < 0.05, **P < 0.005.

Under photopic conditions (3.0 cds/m^2^, background luminance 100 cds/m^2^), a- and b-wave amplitudes, as well as implicit times, revealed no significant differences between treatment groups (i.e., high dose, low dose, sham) or by treatment status (treated versus untreated) at any time point. Regarding longitudinal intragroup changes, a- and b-wave amplitudes and implicit times were unremarkable as well, with two exceptions: high-dose eyes displayed a significant increase in both a- and b-wave implicit times compared to baseline, albeit at different time points: week 4/5 for the a-wave (*P* = 0.0327) and week 12/13 for the b-wave (*P* *=* 0.0053). In addition, sham-injected eyes demonstrated a slight decrease in a-wave (not b-wave) amplitudes at week 12/13 (*P* *=* 0.0428). Figure 3 summarizes photopic ERG results. For the rod-specific stimuli (i.e., 0.0095 cds/m^2^, background luminance: none, 30 minutes of dark adaptation), there were no remarkable changes between treatment groups or in longitudinal analysis regarding both b-wave amplitude and implicit time.

### Ocular Histology

**Figure 4.**
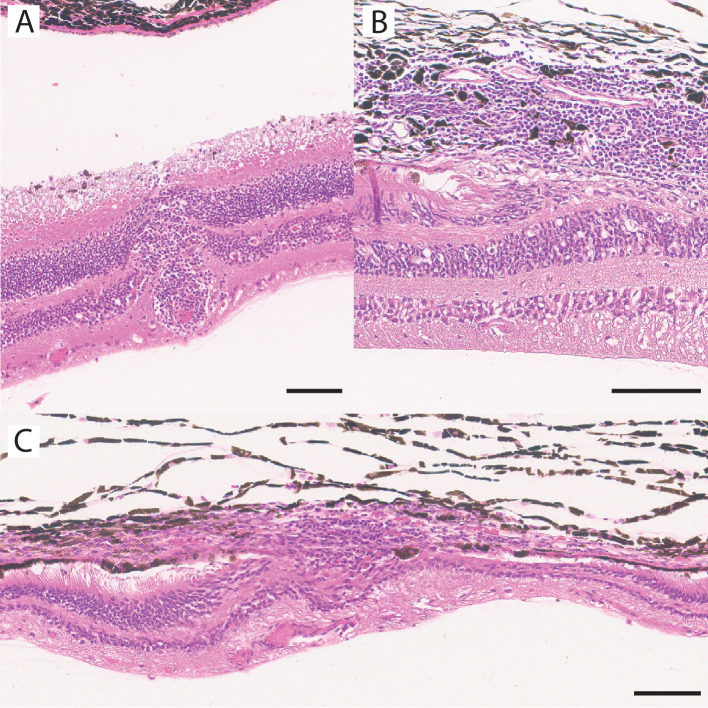
Adverse findings in ocular histology: observed only in high-dose eyes. (**A**) Perivascular mononuclear infiltration. Choroid unremarkable. No relevant atrophy. (**B**) Choroidal mononuclear infiltration. Inner retina unremarkable. Loss of retinal pigment epithelium and photoreceptors. (**C**) Focal full-thickness atrophy with surrounding infiltration. *Black scale bar*: 100 microns.

Sham, low-dose, and intermediate-dose eyes all registered no findings or findings of minimal severity. In the high-dose group, three animals displayed adverse findings. The first affected animal displayed slight focal mononuclear cell infiltration of the choroid associated with focal retinal atrophy. The second animal featured minimal multifocal mononuclear cell infiltration of the choroid as well as minimal multifocal retinal perivascular mononuclear cell infiltration, associated with focal full-thickness retinal atrophy. The third animal also displayed minimal multifocal mononuclear cell infiltration of the choroid and minimal focal retinal perivascular mononuclear cell infiltration, this time associated with a loss of retinal pigment epithelium and photoreceptors. Sample sections from these animals are displayed in Figure 4. Groupwise prevalence (irrespective of severity) of individual ocular histology findings is summarized in the Table and highlights dose dependency of histologic findings.

**Table.** Summary of Microscopic Findings in Retinal Sections Stained With Hematoxylin and Eosin, by Group, With Percentage of Treated Eyes Displaying the Finding

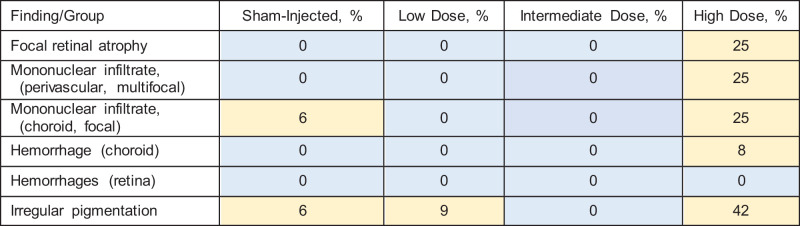				

On histology, clinically significant adverse findings (i.e., especially atrophy) were observed mostly in high-dose eyes. While mild mononuclear infiltration was also observed in one sham-injected eye, frequency and severity of adverse histologic findings were clearly dose dependent.

### OCT and Fundus AF

**Figure 5.**
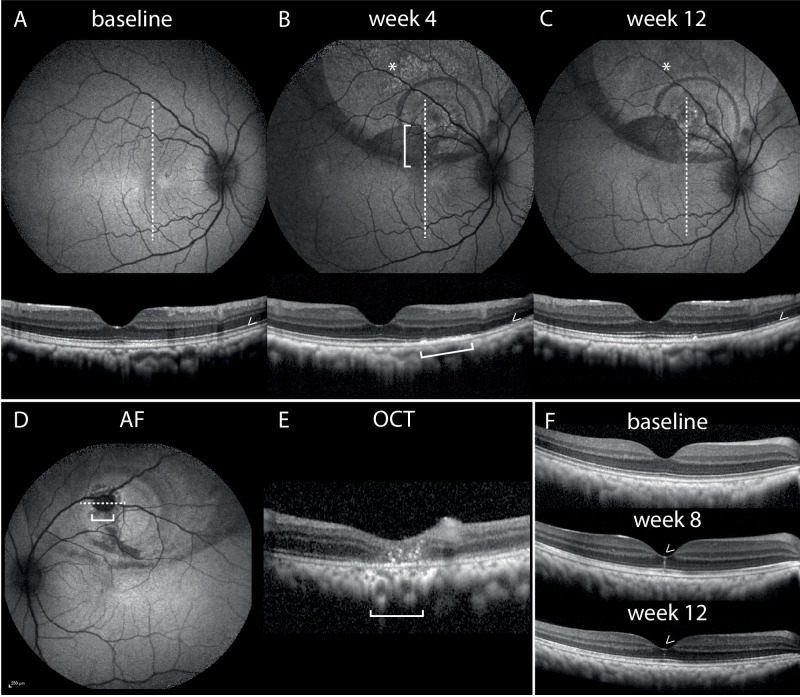
Surgery-related changes in AF and OCT. Sham-treated eye at baseline (**A**), week 4 (**B**), and week 12 (**C**). Atrophy at retinotomy site in a high-dose animal as seen on AF and OCT (**D**, **E**) and findings suggestive of a spontaneously resolved macular hole in a sham-injected animal (**F**). (**A**–**C**) Bleb areas demonstrated pigment irregularities and displacement (*), with excess pigment pooling at the bleb margin. OCT confirms pigment in the subretinal space ([). Areas that were detached during surgery exhibited a transient disruption of the outer retinal (i.e., EZ, ELM) organization (>), which had mostly recovered by week 12. (**D**, **E**) Atrophy at retinotomy site in a high-dose animal ([). (**F**) Findings suggestive of a spontaneously resolved macular hole in a sham-injected animal (>, top to bottom: baseline, week 8, week 12). *Dotted lines* indicate alignment of OCT B-scans. ELM, external limiting membrane; EZ, ellipsoid zone.

On retinal imaging, all treated eyes (including sham) exhibited changes that were attributed to the surgical procedure. The most common was depigmentation of the treated (i.e., “bleb”) area. On OCT, bleb areas (i.e., which had been detached during subretinal application) presented with transient disorganization of outer retinal layers (e.g., external limiting membrane and ellipsoid zone, Figs. 5A–C), which had mostly recovered by week 12. Displaced pigment was found to collect at the bleb margins, resulting in concentrical hyperpigmented rings. The latter were hypo-autofluorescent in AF, with concomitant hyperreflective clumping of pigment at the RPE layer on OCT (Figs. 5A–C). Most likely due to gravitational influence, pigment sedimentation was most pronounced at the inferior bleb border. In addition, three sham-injected eyes displayed changes that were in line with spontaneously resolved macular holes (Fig. 5F). In line with findings of focal atrophy on histology, the injection site was occasionally associated with chorioretinal atrophy. Figures 5D, 5E display one such injection site atrophy in a high-dose eye. In contrast to the abovementioned changes, these atrophic lesions could not be explained by surgery alone and were found to be dose dependent and progressive. A detailed, quantitative analysis of atrophy development across all imaging modalities performed in this study (OCT, AF, FA) has been published recently.^23^

Furthermore, hyperfluorescent foci (HRF) in OCT, as a morphologic surrogate for infiltrating B and T cells and microglia activation, were also found in all eyes of all treatment groups (including sham). Yet, the severity and durability of HRF were found to be dose dependent and most pronounced in high-dose eyes. Data on HRF have already been published by Rodríguez-Bocanegra et al.^24^ and are therefore neither displayed nor discussed further in this publication.

## Discussion

Overall, subretinal injection of 1 × 10^11^ or 5 × 10^11^ vg of rAAV2/8.hPDE6A was well tolerated and elicited no findings, which were considered test article related, adverse, and/or clinically significant.

Clinical findings in slit-lamp examination and fundoscopy were mild and transient in all dose groups. Suturing of the sclerotomies was omitted as they are considered self-healing in human patients, and sutures might have caused more tissue irritation and concomitant digital manipulation by the animals during the recovery period. Yet, the sutureless approach likely contributed to initial ocular hypotony, which was seen in all treated eyes, and in turn led to transient hemorrhaging into the anterior chamber. This was considered a limitation of the nonhuman primate model and is expected not to affect clinical trials.

Regarding inflammatory cellular activity, there was a tendency toward increased inflammation scores in the high-dose cohort. While neither clinically relevant nor statistically significant, this potential increase of overall inflammatory activity was mirrored in ocular histology with mononuclear cellular infiltration being detectable in 25% of high-dose eyes. Of note, longstanding vitritis and/or uveitis, as recently described after gene therapy in humans, was not observed.^25^

ERG results show that a- and b-wave amplitudes were unchanged by administration of rAAV2/8.hPDE6A, irrespective of dose. This indicates that there was not a substantial loss of either photoreceptors (in line with histology) or photoreceptor function. Yet, remarkable results were seen in the high-dose group, which displayed a statistically significant increase in implicit times for both a- and b-waves. While changes were mild and not considered clinically relevant, they were considered both adverse and test article related, especially in light of findings of chorioretinal atrophy at the injection site, which occurred predominantly in high-dose eyes.^23^

Apart from injection site atrophy, various changes were seen on fundus autofluorescence imaging and optical coherence tomography after treatment. Most notably, pigment displacement, irregular pigmentation, and pigment mottling, as seen in most treated eyes, are reported to occur in nonhuman primates after subretinal dosing.^26^ The same is true for the formation of macular holes, outer retinal disorganization, and transient shortening of photoreceptor outer segments, which have all been described after subretinal injections.^26^^,^^27^ All of the above changes were also detected in sham-treated animals and therefore interpreted to be related to the subretinal administration procedure rather than induced by the test article.

In the sham, low-dose, and intermediate-dose groups, findings in ocular histology were minimal and in line with the abovementioned ultrastructural changes introduced by the subretinal injection itself. The only remarkable finding in a non-high-dose eye was a minimal, focal infiltration of mononuclear cells in the retina of one sham-treated eye. This suggests that minimal mononuclear infiltrates alone might arise spontaneously or as a consequence of subretinal injection. However, three high-dose animals displayed evidence of more substantial choroidal and/or perivascular mononuclear cell infiltration, which was associated with the abovementioned focal atrophic changes of the retina and choroid. These changes in the high-dose group were considered test article related and adverse. Given their potential negative impact on visual function, they were also deemed clinically relevant. Considering recent reports of retinal atrophy after treatment with voretigene neparvovec, the development of retinal atrophy in the high-dose cohort of this study emphasizes the need to further investigate the mechanisms of atrophy induction after AAV-based gene therapy. After all, despite methodologic limitations (e.g., healthy NHP versus RPE65-deficient humans, different gene products), these results raise the possibility that atrophic changes after gene therapy might not be limited to voretigene neparvovec.

This study has several limitations. Slit-lamp observations are intrinsically subjective, and due to logistical limitations of the study design, slit-lamp examiners were unmasked. However, cross-validation was performed at week 4 by a third, external, and masked ophthalmologist. It confirmed that all relevant inflammation had receded by week 4. Another limitation is that due to a technical error, rod-specific responses (0.0095 cds/m^2^) were not saved correctly for the a-wave (nonrecoverable data loss). There were no remarkable findings in the corresponding b-wave data. Additional surrogate analysis of dark-adapted cone-rod responses (3 cds/m^2^, background luminance: off, not reported) was in line with photopic ERG results. Therefore, we argue that the lack of a rod-specific a-wave does not preclude the study's main thesis: that the low dose was overall well tolerated, while the high dose was abandoned due to corresponding adverse changes in histology and ERG, as well as on retinal imaging.

In summary, single subretinal injection of rAAV.hPDE6A at a dose of 1 × 10^11^ or 5 × 10^11^ vg was well tolerated, while subretinal injection of a higher dose of 1 × 10^12^ vg was associated with retinal inflammation and atrophic changes, which were considered both test article related and adverse. Therefore, 5 × 10^11^ vg was considered the no observed adverse effect level. Subsequently, a 10-fold lower dose of 5 × 10^10^ vg was chosen as the starting dose for the ongoing phase I/II clinical trial for retinitis pigmentosa due to *PDE6A* mutations.

## References

[1] Reichel FF, Seitz I, Wozar F, et al. Development of retinal atrophy after subretinal gene therapy with voretigene neparvovec. *Br J Ophthalmol*. 2023; 107(9): 1331–1335. 35609955 10.1136/bjophthalmol-2021-321023

[2] Kolesnikova M, Lima de Carvalho JR Jr, Parmann R, et al. Chorioretinal atrophy following voretigene neparvovec despite the presence of fundus autofluorescence. *Mol Genet Genomic Med*. 2022; 10(11): e2038. 36225124 10.1002/mgg3.2038 PMC9651599

[3] Boye SL, Choudhury S, Crosson S, et al. Novel AAV44.9-based vectors display exceptional characteristics for retinal gene therapy. *Mol Ther*. 2020; 28(6): 1464–1478. 32304666 10.1016/j.ymthe.2020.04.002 PMC7264435

[4] Pavlou M, Schön C, Occelli LM, et al. Novel AAV capsids for intravitreal gene therapy of photoreceptor disorders. *EMBO Mol Med*. 2021; 13(4): e13392. 33616280 10.15252/emmm.202013392 PMC8033523

[5] Chien Y, Hsiao YJ, Chou SJ, et al. Nanoparticles-mediated CRISPR-Cas9 gene therapy in inherited retinal diseases: applications, challenges, and emerging opportunities. *J Nanobiotechnol*. 2022; 20(1): 511. 10.1186/s12951-022-01717-x PMC9719668 36463195

[6] Kantor A, McClements ME, Peddle CF, et al. CRISPR genome engineering for retinal diseases. *Prog Mol Biol Transl Sci*. 2021; 182: 29–79. 34175046 10.1016/bs.pmbts.2021.01.024

[7] Bonillo M, Pfromm J, Fischer MD. Challenges to gene editing approaches in the retina. *Klin Monbl Augenheilkd*. 2022; 239(3): 275–283. 35316854 10.1055/a-1757-9810

[8] Ren D, Fisson S, Dalkara D, Ail D. Immune responses to gene editing by viral and non-viral delivery vectors used in retinal gene therapy. *Pharmaceutics*. 2022; 14(9): 1973. 36145721 10.3390/pharmaceutics14091973 PMC9502120

[9] Wozar F, Seitz I, Reichel F, Fischer MD. Importance of nonhuman primates as a model system for gene therapy development in ophthalmology. *Klin Monbl Augenheilkd*. 2022; 239(3): 270–274. 35189657 10.1055/a-1777-5033

[10] Boughman JA, Conneally PM, Nance WE. Population genetic studies of retinitis pigmentosa. *Am J Hum Genet*. 1980; 32(2): 223–235. 7386458 PMC1686021

[11] Littink KW, van den Born LI, Koenekoop RK, et al. Mutations in the EYS gene account for approximately 5% of autosomal recessive retinitis pigmentosa and cause a fairly homogeneous phenotype. *Ophthalmology*. 2010; 117(10): 2026–2033, 33e1-7. 20537394 10.1016/j.ophtha.2010.01.040

[12] Audo I, Sahel JA, Mohand-Said S, et al. EYS is a major gene for rod-cone dystrophies in France. *Hum Mutat*. 2010; 31(5): E1406–E1435. 20333770 10.1002/humu.21249

[13] Seyedahmadi BJ, Rivolta C, Keene JA, Berson EL, Dryja TP. Comprehensive screening of the USH2A gene in Usher syndrome type II and non-syndromic recessive retinitis pigmentosa. *Exp Eye Res*. 2004; 79(2): 167–173. 15325563 10.1016/j.exer.2004.03.005

[14] Avila-Fernandez A, Cantalapiedra D, Aller E, et al. Mutation analysis of 272 Spanish families affected by autosomal recessive retinitis pigmentosa using a genotyping microarray. *Mol Vis*. 2010; 16: 2550–2558. 21151602 PMC3000238

[15] den Hollander AI, Davis J, van der Velde-Visser SD, et al. CRB1 mutation spectrum in inherited retinal dystrophies. *Hum Mutat*. 2004; 24(5): 355–369. 15459956 10.1002/humu.20093

[16] McLaughlin ME, Ehrhart TL, Berson EL, Dryja TP. Mutation spectrum of the gene encoding the beta subunit of rod phosphodiesterase among patients with autosomal recessive retinitis pigmentosa. *Proc Natl Acad Sci USA*. 1995; 92(8): 3249–3253. 7724547 10.1073/pnas.92.8.3249 PMC42143

[17] Dryja TP, Rucinski DE, Chen SH, Berson EL. Frequency of mutations in the gene encoding the alpha subunit of rod cGMP-phosphodiesterase in autosomal recessive retinitis pigmentosa. *Invest Ophthalmol Vis Sci*. 1999; 40(8): 1859–1865. 10393062

[18] Wert KJ, Davis RJ, Sancho-Pelluz J, Nishina PM, Tsang SH. Gene therapy provides long-term visual function in a pre-clinical model of retinitis pigmentosa. *Hum Mol Genet*. 2013; 22(3): 558–567. 23108158 10.1093/hmg/dds466 PMC3542865

[19] Schon C, Sothilingam V, Muhlfriedel R, et al. Gene therapy successfully delays degeneration in a mouse model of PDE6A-linked retinitis pigmentosa (RP 43). *Hum Gene Ther*. 2017; 28(12): 1180–1188. 29212391 10.1089/hum.2017.156

[20] Mowat FM, Occelli LM, Bartoe JT, et al. Gene therapy in a large animal model of PDE6A-retinitis pigmentosa. *Front Neurosci*. 2017; 11: 342. 28676737 10.3389/fnins.2017.00342 PMC5476745

[21] Occelli LM, Schon C, Seeliger MW, et al . Gene supplementation rescues rod function and preserves photoreceptor and retinal morphology in dogs, leading the way towards treating human PDE6A-retinitis pigmentosa. *Hum Gene Ther*. 2017; 28(12): 1189–1201. 29212382 10.1089/hum.2017.155

[22] Petersen-Jones SM, Occelli LM, Winkler PA, et al. Patients and animal models of CNGbeta1-deficient retinitis pigmentosa support gene augmentation approach. *J Clin Invest*. 2018; 128(1): 190–206. 29202463 10.1172/JCI95161 PMC5749539

[23] Seitz IP, Wozar F, Ochakovski GA, et al. Dose-dependent progression of chorioretinal atrophy at the injection site after subretinal injection of rAAV2/8 in non-human primates. *Ophthalmol Sci*. 2024; 4(5): 100516. 38881604 10.1016/j.xops.2024.100516 PMC11179412

[24] Rodríguez-Bocanegra E, Wozar F, Seitz IP, et al. Longitudinal evaluation of hyper-reflective foci in the retina following subretinal delivery of adeno-associated virus in non-human primates. *Transl Vis Sci Technol*. 2021; 10(6): 15. 10.1167/tvst.10.6.15 PMC8114007 34111260

[25] Kessel L, Christensen UC, Klemp K. Inflammation after voretigene neparvovec administration in patients with RPE65-related retinal dystrophy. *Ophthalmology*. 2022; 129(11): 1287–1293. 35760216 10.1016/j.ophtha.2022.06.018

[26] Nork TM, Murphy CJ, Kim CB, et al. Functional and anatomic consequences of subretinal dosing in the cynomolgus macaque. *Arch Ophthalmol*. 2012; 130(1): 65–75. 21911651 10.1001/archophthalmol.2011.295 PMC3254795

[27] Aleman TS, Huckfeldt RM, Serrano LW, et al. Adeno-associated virus serotype 2-hCHM subretinal delivery to the macula in choroideremia: two-year interim results of an ongoing phase I/II gene therapy trial. *Ophthalmology*. 2022; 129(10): 1177–1191. 35714735 10.1016/j.ophtha.2022.06.006

